# Neuroendocrine differentiation in a case of cervical cancer

**Published:** 2010-07-28

**Authors:** Rashed Mona Mohamed, Bekele Alemayehu

**Affiliations:** 1 Department of Pathology, General Organization of Teaching Hospitals and Institutes, Egypt; 2 Department of Pathology, Jimma University, Ethiopia

**Keywords:** Cervical cancer, cervix, chromogranine A, small cell carcinoma

## Abstract

Neuroendocrine neoplasms may occur in the uterine cervix, although rarely; it accounts for 0.5-1% of all malignant tumors of the uterine cervix. A case report of an Ethiopian female presented at the Gynecology Out-Patient Clinic at Jimma University Hospital, complaining from irregular vaginal bleeding over the previous three months. Clinically there was a cauliflower cervical mass; histopathologically it was formed of sheets of small cell tumor; that further showed neuroendocrine differentiation, as demonstrated by chromogranin-A positivity. It is important to differentiate small cell carcinoma from other malignant tumors of the uterine cervix. Morphological features play an important role in making a diagnosis and the immunohistochemistry study can offer an additional useful assistance.

## Introduction

Neuroendocrine neoplasm may occur in the uterine cervix; it accounts for 0.5-1% of all malignant tumors of the uterine cervix [1,2]. It is generally accepted that the integration of HPV into the host genome is the single most important event in evolution of cervical carcinomas [3]. Almost all neuroendocrine carcinomas of the cervix are associated with HPV 18 or seldom HPV 16 [4]. Neuroendocrine carcinomas most likely develop from neuroendocrine cells occurring in the normal endocervix or from stimulated multipotential reserve cells of the endocervical epithelium undergoing neuroendocrine metaplasia and hyperplasia [5]. Neuroendocrine tumors of uterine cervix are divided into small and large cell type as well as carcinoid and atypical carcinoid [3]. Small cell neuroendocrine carcinoma of the uterine cervix is a rare tumor with a highly aggressive clinical course and poor prognosis due to the high frequency of lymph node involvement at an early stage [4,6]. It is important to differentiate small cell carcinoma of the uterine cervix from other malignant tumors of the cervix [4]. Morphological features, cytopathology and histopathology as well as the immunocytochemistry studies play important roles in making an accuratediagnosis [2,7].

## Patient and case report

An Ethiopian female patient presented at the Gynecology Out-Patient Clinic at Jimma University Hospital, she was 42 years old; complaining of irregular vaginal bleeding over the previous three months. On clinical examination there was a cervical cauliflower mass about 4x4 cm, covering the entire cervical surface. The cervical mass was bleeding on touch. The pelvic ultrasound revealed a bulky cervix with a heterogeneous echo-pattern; moderate pelvic fluid collections were noticed. The patient was serologically negative for HIV. Total abdominal hysterectomy with bilateral salpingo-oophorectomy was performed. The uterus was of normal size with no recognizable tumors either grossly of microscopically. The lymph nodes couldn’t be assessed. The tumor was staged as Stage IB2: T1b2 N0 M0

**Figure 1:**
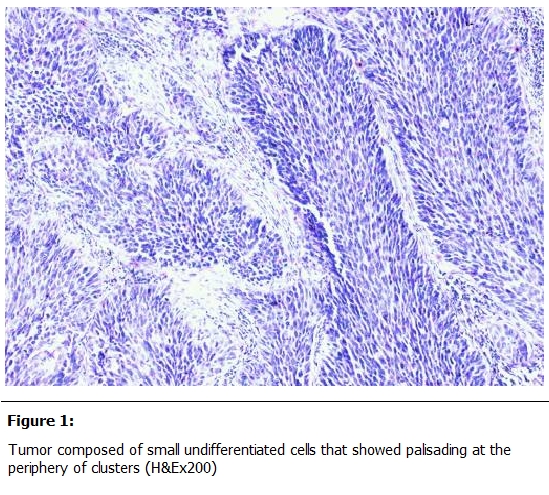
Tumor composed of small undifferentiated cells that showed palisading at the periphery of clusters (H&Ex200)

**Figure 2:**
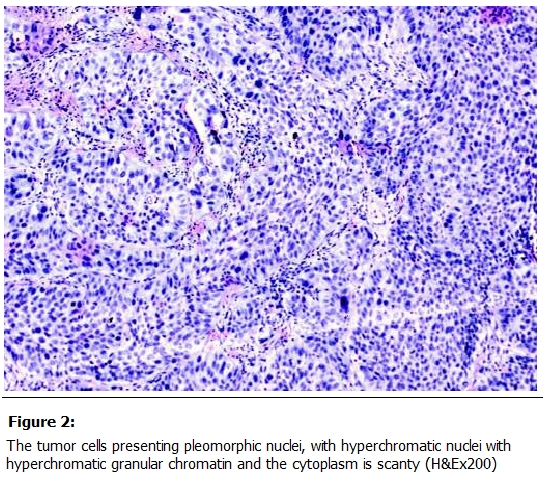
The tumor cells presenting pleomorphic nuclei, with hyperchromatic nuclei with hyperchromatic granular chromatin and the cytoplasm is scanty (H&Ex200)

Histo-pathological examination of the cervical mass revealed tumor cells arranged into lobules and solid sheets, the tumor was composed of small undifferentiated cells that showed palisading at the periphery of clusters (figure 1). The tumor cells presented pleomorphic nuclei, with hyperchromatic nuclei, hyperchromatic granular chromatin and the cytoplasm was scanty. Moreover the nuclear-cytoplasmic ratio was high and there was an evidence of increased mitosis as well as apoptotic changes (figure 2).

**Figure 3:**
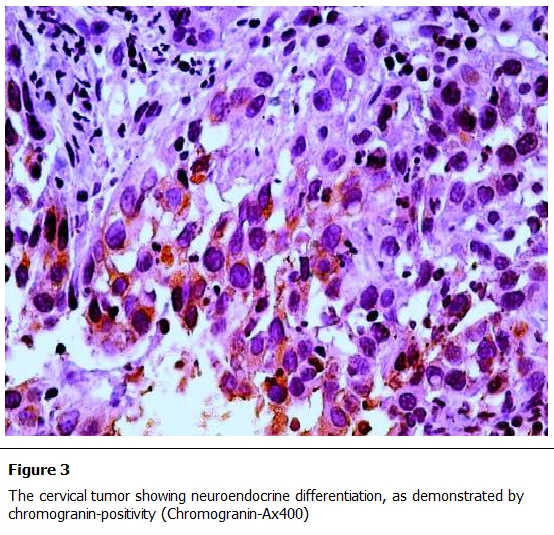
The cervical tumor showing neuroendocrine differentiation, as demonstrated by chromogranin-positivity (Chromogranin-Ax400)

Immuno-histochemical study was done aiming to recognize neuroendocrine differentiation of the tumor. The paraffin-embedded sections of the cervix were immune-histochemically characterized with antibody to chromogranin-A, the bound antibodies were detected by a standard avidinbiotin complex method with a peroxidase and diaminobenzidine color development system. The cervical tumor showed neuroendocrine differentiation, as demonstrated by chromogranin-A positivity (figure 3).

The diagnosis was pathologically reported as “Small Cell Type of Neuroendocrine Cancer of Uterine Cervix” and the patient was referred to the Oncology Department at Addis Ababa University for chemo-radiotherapy.

## Discussion

Small cell carcinomas of neuroendocrine origin are considered to be the poorly differentiated variety of carcinoid tumors; it is a rare tumor with a highly aggressive clinical course and poor prognosis due to the high frequency of lymph node involvement at an early stage [7]; thus neuroendocrine differentiation is an indicator of poor prognosis [8].

The normal endocervix contains as many as 20% of argyophilic cells resembling endocrine cells; cervical neuroendocrine tumor formation can arise from these cells [9]. Savargaonkar et al reported that neuroendocrine differentiation was present in 20.9% of his series study of cervical carcinoma [5].

Clinically, abnormal vaginal bleeding is the most commonly reported symptoms. In spite of the neuroendocrine origin of this tumor, the clinical carcinoid syndrome in the neuroendocrine tumors of the cervix is very unusual [10]; but on the other hand, a primary carcinoid tumor other than the cervix, with direct secretion of its mediators into the systemic circulation could be responsible for the carcinoid syndrome [9].

The distinction of squamous, glandular and neuroendocrine carcinomas of the cervix is clinically significant for at least two reasons. First, a poorly differentiated carcinoma of glandular origin, even with early invasion, is likely to have a worse prognosis than a similar squamous tumor [11]. Second, neuroendocrine carcinomas are inherently more aggressive than their squamous counterparts and are managed with different protocols [12]. Expression of chromogranin A, synaptophysin, and various other proteins involved in the formation of neurosecretory granules or CD 56, a neural cell adhesion molecule, can be used as markers of neuroendocrine differentiation, as in neuroendocrine carcinomas of other organs [13]. Neuroendocrine carcinomas of the cervix are regarded as highly aggressive tumors [14] with subclinical hematogenous and lymphatic metastases frequently even in early disease. Neuroendocrine features in poorly differentiated carcinomas of the cervix indicate a poor outcome [15]. Sixty-five percent of patients with cervical non-small cell neuroendocrine carcinomas die within 3 yr of diagnosis [1].

## Conclusion

We concluded that it is important to differentiate small cell carcinoma from other malignant tumors of the uterine cervix. The diagnosis of small cell neuroendocrine carcinoma depends on the combination of light microscopy and immunohistochemistry. Morphological features play an important role in making a diagnosis and the immunohistochemistry study can offer an additional useful assistance.

## Competing interests

The authors declare no competing interests.

## Authors’ contributions

**M.Rashed** was the pathologist responsible for confirming the diagnosis of the case by the immunohistochemistry study. **A. Bekele** was the first to see the case as neuroendocrine carcinoma and both shared the ideas in reporting this case report.
